# Antibodies against *Rickettsia* spp. in Hunters, Germany

**DOI:** 10.3201/eid1412.080229

**Published:** 2008-12

**Authors:** Andreas Jansen, Bernard La Scola, Didier Raoult, Michael Lierz, Ole Wichmann, Klaus Stark, Thomas Schneider

**Affiliations:** Robert Koch Institute, Berlin, Germany (A. Jansen, O. Wichmann, K. Stark); Faculté de Médecine, Marseille, France (B. La Scola, D. Raoult); Free University of Berlin, Berlin (M. Lierz); Charité, Berlin (T. Schneider)

**Keywords:** Rickettsiae, epidemiology, arthropod-borne, letter

**To the Editor:** A number of emerging *Rickettsia* species have been recently described (*1*). One of these, *R. helvetica*, was first isolated in Switzerland in 1979 and was implicated in perimyocarditis and nonspecific febrile disease in humans (*2*–*5*). PCR showed its prevalence in 1,187 *Ixodes ricinus* ticks in southern Germany to be 8.9% (*6*). This finding raises the question whether autochthonous transmission of rickettsiae to humans may occur in Germany. To help answer this question, we conducted a cross-sectional study of the presence of antibodies against *Rickettsia* spp. in a population in Germany presumably exposed to ticks.

On February 4–5, 2006, we used convenience sampling to enroll 286 hunters at a national hunting fair in Dortmund, Germany. All study participants gave written, informed consent. The Ethics Committee of the Charité approved the study.

Every participant completed a standardized questionnaire. Serum samples were collected from all hunters and analyzed by immunofluorescence assay for 9 *Rickettsia* species (*R. conorii, R. slovaca, R. helvetica*, *R. massiliae, R. mongolitimonae, R. israelensis, R. aeschlimannii, R. felis,* and *R. typhi*) as described previously (*7*). Odds ratios (ORs) and 95% confidence intervals (CIs) were calculated by using SPSS software version 14 (SPSS, Inc., Chicago, IL, USA). We considered p<0.05 to be significant.

Of the 286 hunters, 252 (88.1%) were male; median age was 46 years (range 17–79 years). Positive antibody titers (immunoglobulin [Ig] G, IgM, or both) against any *Rickettsia* spp. were found for 26 (9.1%) hunters (95% CI 6.2–13.0). Antibodies against different *Rickettsia* spp. were found for 18 hunters; species-specific antibodies against *R. helvetica* were found for 2 hunters and against *R. aeschlimannii* for 6 (Table). Seropositive and seronegative hunters did not differ significantly with respect to sex, age, and total years of hunting. Neither hunting nor traveling in a foreign country within the past 5 years was significantly associated with seropositivity. Neither of the 2 hunters with *R. helvetica–*specific antibody titers had traveled outside Germany in the 5 years before the study, but 3 of the 6 hunters with specific titers against *R. aeschlimannii* had traveled and hunted in countries with unknown endemicity for *R. aeschlimannii* (Russia, Romania, Namibia). A total of 212 (74.1%) hunters had received at least 1 tick bite in the year before the study; median was 4 tick bites/year. Living in the southern parts of Germany (below 50°N) was significantly related to seropositivity (OR 4.1, 95% CI 1.3–12.3, p = 0.02). Although the 26 persons with positive serologic results for *Rickettsia* spp. reported arthralgia with higher frequency than did seronegative persons (50% vs. 37%, respectively), their reports of arthralgia and of other clinical signs did not differ significantly: temperature >38.5°C (8% vs. 2%), enlarged lymph nodes (12% vs. 9%). No seropositive hunter reported having had an eschar.

**Table:** Positive immunofluorescence assay results for antigens to 9 *Rickettsia* spp. in 26 hunters, Germany, 2006*

Hunter no.	IgG/IgM								
	*R.* *conorii*	*R.* *slovaca*	*R.* *helvetica*	*R.* *massiliae*	*R.* *mongolitimonae*	*R.* *israelensis*	*R.* *aeschlimannii*	*R.* *felis*	*R.* *typhi*
106			0/64						
109	**64/128**	**64/128**	**64/128**	**64/128**	**64/128**		**32/32**	**64/0**	**0/128**
111		**0/32**	**0/64**	**0/32**					
113			**0/128**						**0/64**
115		**0/32**	**0/128**	**0/32**	**0/32**	**0/32**	**0/64**	**0/32**	**0/64**
117			**0/32**	**0/32**	**0/32**				**0/32**
124		**0/32**	**0/64**	**0/32**	**0/32**	**0/32**	**0/32**		**0/64**
127			**0/128**						**0/64**
130	**0/64**	**0/64**	**0/128**	**0/32**		**0/32**	**0/64**		**0/64**
131			256/0					**0/64**	
142	**0/64**	**0/64**	**0/64**	**0/64**	**0/64**				
148		**0/64**	**0/64**	**0/64**	**0/32**				
151	**0/64**	**0/64**	**0/64**	**0/64**	**0/64**				
160		**0/32**	**0/32**	**0/32**	**0/32**				
161		**0/64**	**0/64**	**0/32**	**0/32**				
182							128/0		
185							64/64		
224	**64/64**	**64/64**	**64/64**	**64/64**	**64/64**	**64/64**	**64/64**	**64/64**	**64/64**
230		**0/32**		**0/32**	**0/32**				
233		**0/32**	**0/32**	**0/32**	**0/32**	**0/32**	64/16		
236							64/16		
237							64/32		
277			**64/32**				**64/32**		
281							64/32		
285		**0/32**	**0/32**				**0/32**	**0/32**	**0/32**
297		**0/32**	**0/32**	**0/32**	**0/32**	**0/32**	**0/32**	**0/32**	**0/32**

*All specimens were tested for all antigens. **Boldface** indicates nonspecific titers. Cutoff titers for seropositivity (immunoglobulin [Ig] G or IgM) were 128/64 for *R. conorii* and 64/32 for other antigens (*8*). A rickettsial antigen was considered to represent the agent of infection when cross-reactions were absent or when titers of IgG or IgM antibody against this antigen were >2 serial dilutions higher than titers of IgG or IgM antibody against other rickettsial antigens.

This study provides data for Germany on the seroprevalence of *Rickettsia* spp. in persons highly exposed to ticks. Our results suggest that *Rickettsia* spp. are endemic to southern Germany and may cause autochthonous infections. Although most seropositive hunters exhibited reactivity to several rickettsial antigens, some had species-specific titers for *R. helvetica*. Six hunters exhibited specific reactivity to *R. aeschlimannii*. Serologic cross-reactions are frequently noted among spotted fever group rickettsiae, and 1 of the best indicators of species identity remains the geographic origin of the infection (*7*). Until now, *R. aeschlimannii* had not been detected in Germany or neighboring countries. We therefore suggest that the specific titers against *R. aeschlimannii* in our study population may be partly related to traveling or hunting abroad and that the observed seroprevalence for other rickettsial species is most likely caused by *R. helvetica*, or, alternatively, by *R. monacensis*, which was recently isolated from a tick in the English Garden in Munich (*9*). Cutoff titers for IgM and IgG were chosen to achieve a specificity >98%; sensitivity varied between different rickettsial antigens. However, if we assume a sensitivity of only 50% (with a prevalence of 9.1%), the positive predictive value of our test would still be 74%. In addition, a test with high specificity and low sensitivity underestimates the true seroprevalence; the proportion of seropositive hunters in our study group is likely higher.

Although hunters with positive immunofluorescence assay results reported having had symptoms compatible with rickettsioses more frequently than did seronegative hunters, these differences were not significant. A similar situation has been noted for persons who were tested for antibodies against *Borrelia burgdorferi* and human granulocytic anaplasmosis; the findings may reflect the mild and poorly defined clinical picture that is typical for each of these diseases (*10*).

To conclude, we report the presence of *Rickettsia* spp. antibodies in a high-risk group from Germany. Final proof that human rickettsiosis occurs in Germany, however, will require the isolation of the agent from patients.
